# Supplementary figures and images for: Gradient in cytoplasmic pressure in germline cells controls overlying epithelial cell morphogenesis
Source: PLoS Biol. 2020 Nov 30;18(11):e3000940. doi: 10.1371/journal.pbio.3000940 (PMC7703951; doi:10.1371/journal.pbio.3000940)

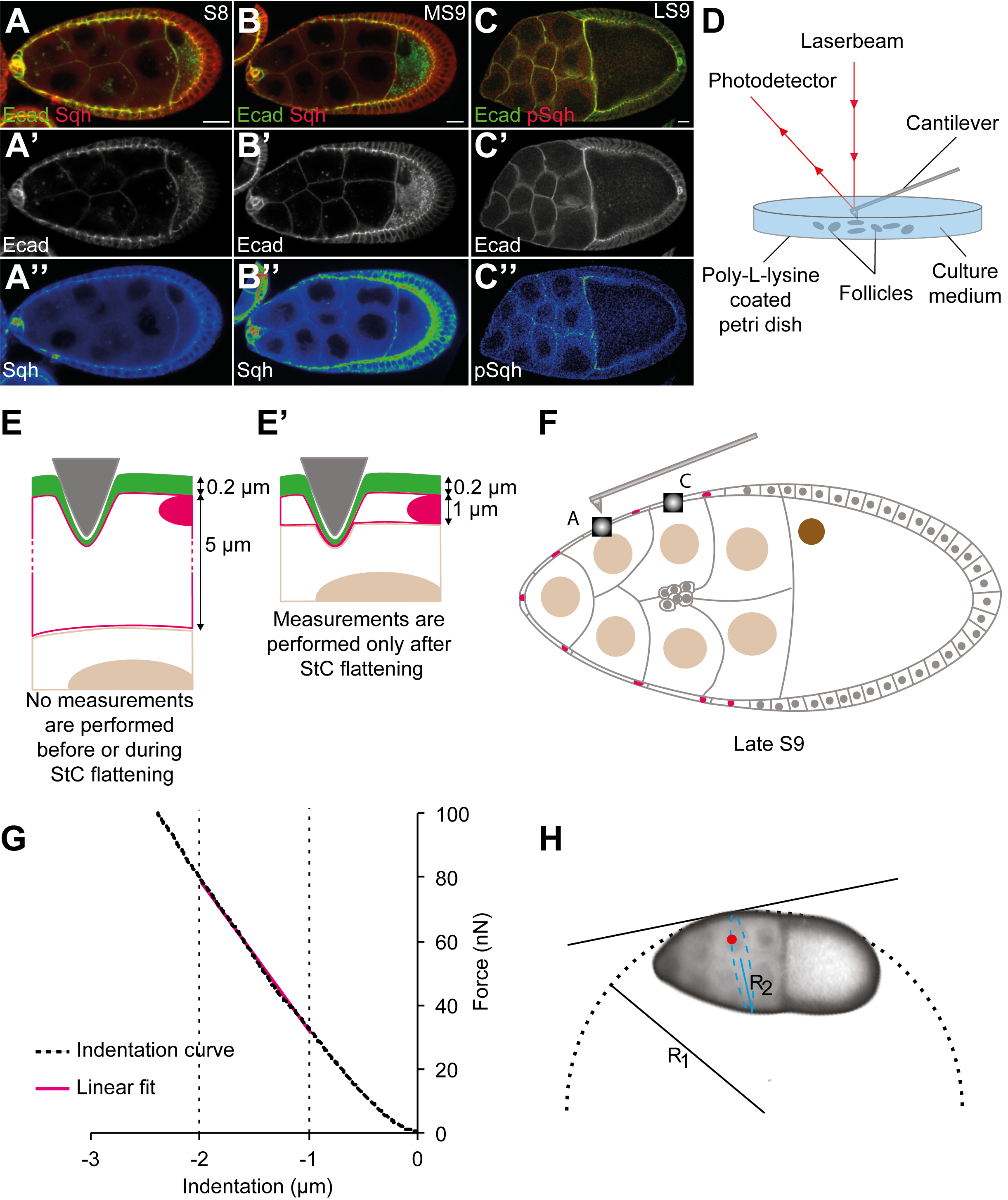

Supplement: S1 Fig — (A-C) Sections through WT live follicles (A-B) or fixed follicles (C). (D) Diagram of the setup used to perform AFM measurements. Living follicles are stuck to a poly-L-lysine–coated petri dish filled with culture medium. The AFM comprises a cantilever with a tip that is used to probe the follicles. The bending of the lever when it encounters the follicle is detected by a photodiode that captures changes in a laser beam reflected onto the upper face of the cantilever. (E) Schematic representation of the probed area: the tip deforms the basement membrane (green) and the StC (red). In StC that have already flattened (E’), the underlying nurse cells (brown) are also deformed, allowing measurements. (F) Schematic representation of a LS9 follicle and of the cantilever. At this stage, only two regions were probed: above the anterior (“A”) and central (“C”) nurse cells. The posterior nurse cells are probed only in S10 follicles. For each region, a 10 × 10 matrix is measured, with indentation points spaced 100 nm apart. (G) Force–indentation depth curve obtained on a follicle with a pyramidal probe tip. The curve is fitted using the linear model (red line) to obtain the elastic modulus. Only the zone of interest (−1 to −2 μm) is fitted. (H) Schematic representation of the geometric measurements taken at areas where inner pressure was measured with AFM. Two circles are drawn to fit either the entire follicle (dotted black line) or the AFM-probed area (dotted blue line at red dot), and the two radii, R1 and R2, are used to calculate inner pressure (see Methods). Scale bar: 20 μm. AFM, atomic force microscope; LS9, late S9; S, stage; StC, stretched cell; WT, wild type. (TIF) [file pbio.3000940.s004.tif]

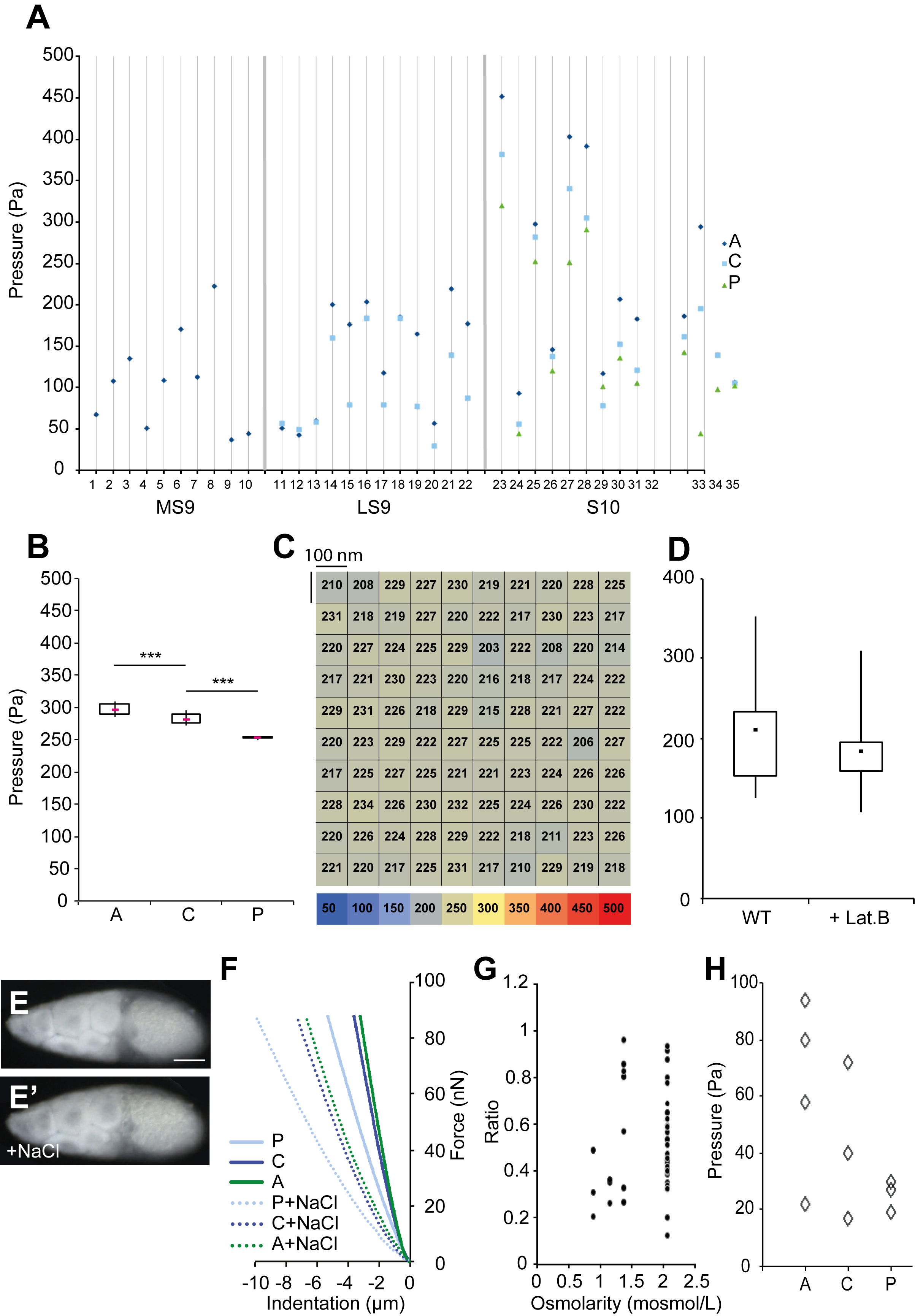

Supplement: S2 Fig — (A) Inner pressures of anterior (“A”), central (“C”), and posterior (“P”) NCs in individual WT follicles at S9 and S10. (B) Box and whisker plots of the 100 measurements taken in anterior (“A”), central (“C”), and posterior (“P”) NCs from a S10 follicle. (C) Color-coded representation of the 100 measurements in a single probed region of a WT S10 follicle. The color-coded scale is shown. (D) AFM measurements of inner pressure in WT LS9 and S10A follicle before and after Latrunculin B treatment (n = 10). (E) WT S10 follicle before (E) and after (E’) NaCl treatment. (F) Force–indentation depth curves from anterior, central, and posterior NCs of a WT S10 follicle before (solid lines) and after (dotted lines) NaCl treatment. (G) Fold-change of inner pressure following osmotic treatment shown as a function of solution osmolarity (NaCl 0.5 M in water–NaCl 0.5 M in PBS–Sorbitol 1 M in PBS–NaCl 1 M in PBS from the lowest to the highest osmolarity). No significant influence of the position of the NC (anterior, central, or posterior) has been observed. (H) AFM measurements of inner pressure in anterior, central, or posterior NCs after collagenase treatment. Scale bar: 50 μm. Data for graphs (A), (B), (D), (F), (G), and (H) can be found in the S1 Data file. AFM, atomic force microscope; LS9, late S9; NC, nurse cell; S, stage; WT, wild type. (TIF) [file pbio.3000940.s005.tif]

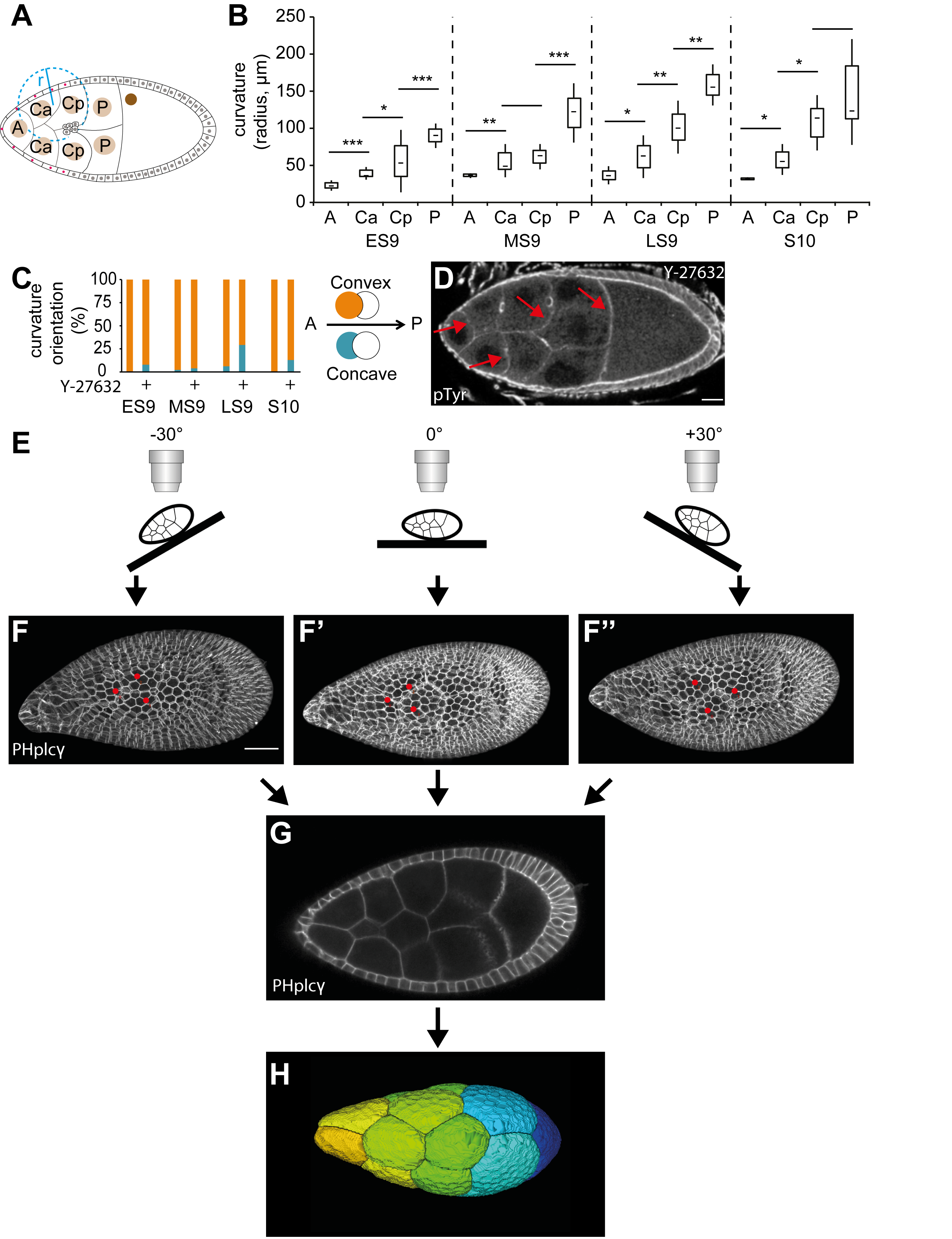

Supplement: S3 Fig — (A) Schematic representation of a WT S9 follicle representing the method used to measure NC curvature in fixed follicles: circles (blue dotted line) are apposed to fit a particular NC membrane and the radius (r) is measured. (B) Box and whisker plots of radii of the membrane curvature for anterior (“A”), central (“C,” both the anteriormost “Ca” and the posteriormost “Cp”), and posterior (“P”) NCs in WT ES 9 to S10 follicles (n = 98 cells for ES9; 42 for MS9; 25 for LS9; and 15 for S10). (C) Percentage of convex (orange) and concave (blue) NC posterior membrane curvatures at different stages in presence or not of the ROCK inhibitor (Y-27632) for WT follicles. (D) WT follicle after addition of ROCK inhibitor. (E) Schematic representation of sample acquisitions for the MARS method. (F) The 2D surface projections of a WT S8 follicle imaged at three different angles. Reference points (red dots) are used to fuse the stacks. (G) A mid-Z-slice through a 3D reconstructed follicle where the three individual image stacks were fused into a single high-resolution stack. (H) The 3D segmentation of the reconstructed follicle (G) showing only the germline cells. Scale bar: 20 μm. Data for graphs (B) and (C) can be found in the S1 Data file. ES9, early S9; LS9, late S9; MS9, mid S9; NC, nurse cell; S, stage; WT, wild type. (TIF) [file pbio.3000940.s006.tif]

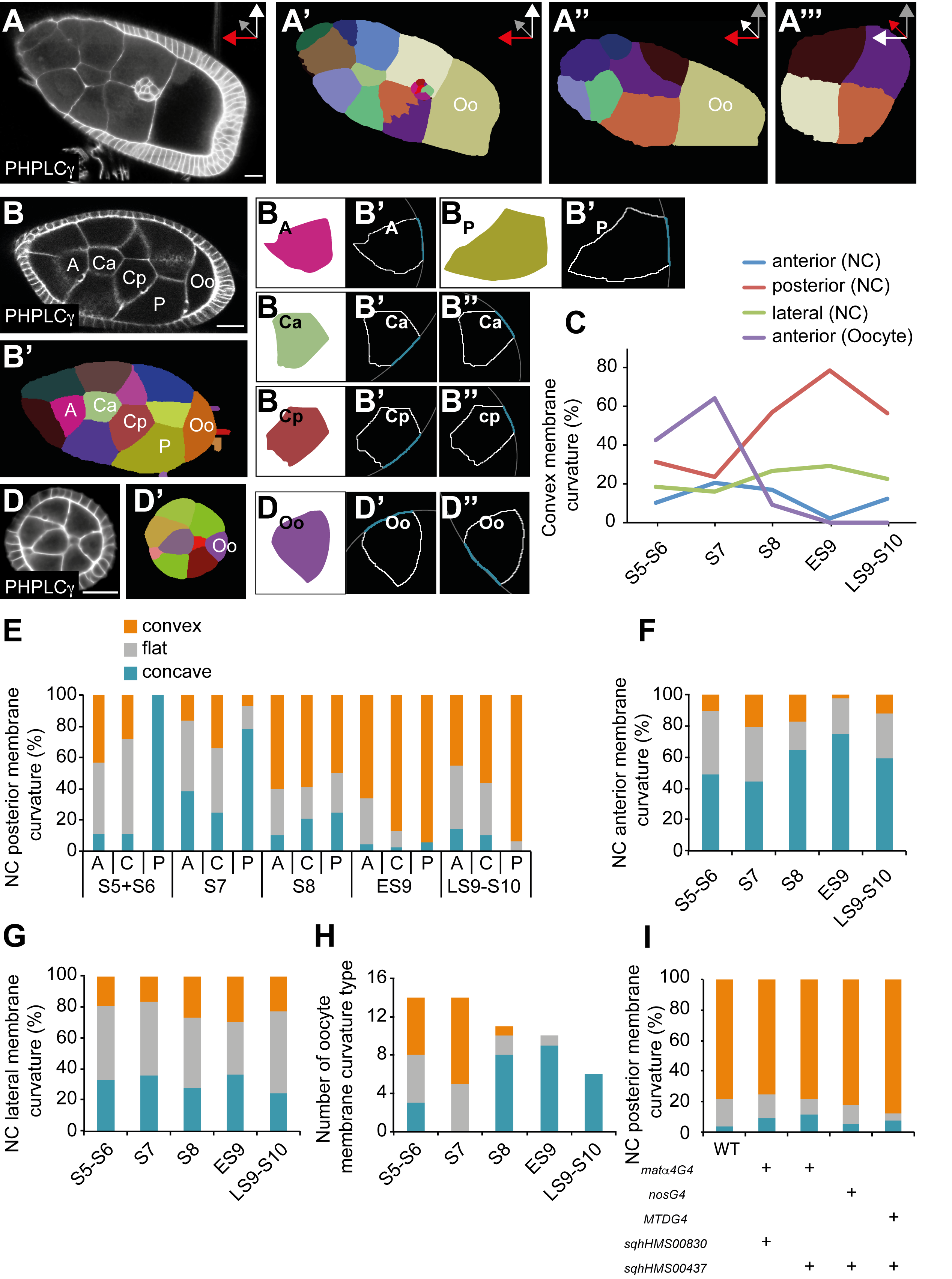

Supplement: S4 Fig — (A) A mid-Z-slice through a 3D reconstructed mid S9 follicle. (A’-A”‘) Segmented germline cells visualized from the z (A’), y (A”), or x (A”‘) axis. (B) The S8 follicle presented in Fig 2G viewed at a different z section, along with the corresponding 3D segmentation (B’). BA, BCa, BCp, and BP show to slices with the largest area in the 3D segmentation of the NC under study, which are labeled as in Fig 2G. The (B’) panels present the outlines (solid white lines) of the same cells, as well as the circles (dotted white lines) fitting the posterior membranes (blue lines). (C) Percentage of anterior (“A”), posterior (“P”), and lateral convex NC membranes in WT S5 to S10 follicles. (D) Slice through a 3D reconstructed S6 follicle along with the 3D segmented image of its germline cells (D’). The slice with the largest area within the segmented oocyte in the z axis is shown (DOo) alongside its outline (solid white line) and the circles fitting the anterior membranes (blue line) (D’Oo to D”Oo). (E) Percentage of convex (orange), flat (gray), and concave (blue) NC posterior membrane curvatures in function of the position of the NCs along the A/P axis (anterior [“A”], central [“C”], and posterior [“P”]) in WT S5 to S10 follicles (n comprised between 9 and 49 cells). (F-H) Percentage (F, G) or number (H) of convex (orange), flat (gray), or concave (blue) curvatures of anterior NC membranes (F, n > 60), lateral NC membranes (G, n > 100), or anterior oocyte membranes (H) in WT S5 to S10 follicles. Two or three follicles were reconstructed and segmented per stage. (I) Percentage of convex (orange), flat (gray), and concave (blue) NC posterior membrane curvatures at different follicular stages in WT or in follicles with reduced sqh germline activity (n > 167). Scale bar: 20 μm. Data for graphs (C), (E), (F), (G), (H), and (I) can be found in the S1 Data file. NC, nurse cell; S, stage; sqh, spaghetti squash; WT, wild type. (TIF) [file pbio.3000940.s007.tif]

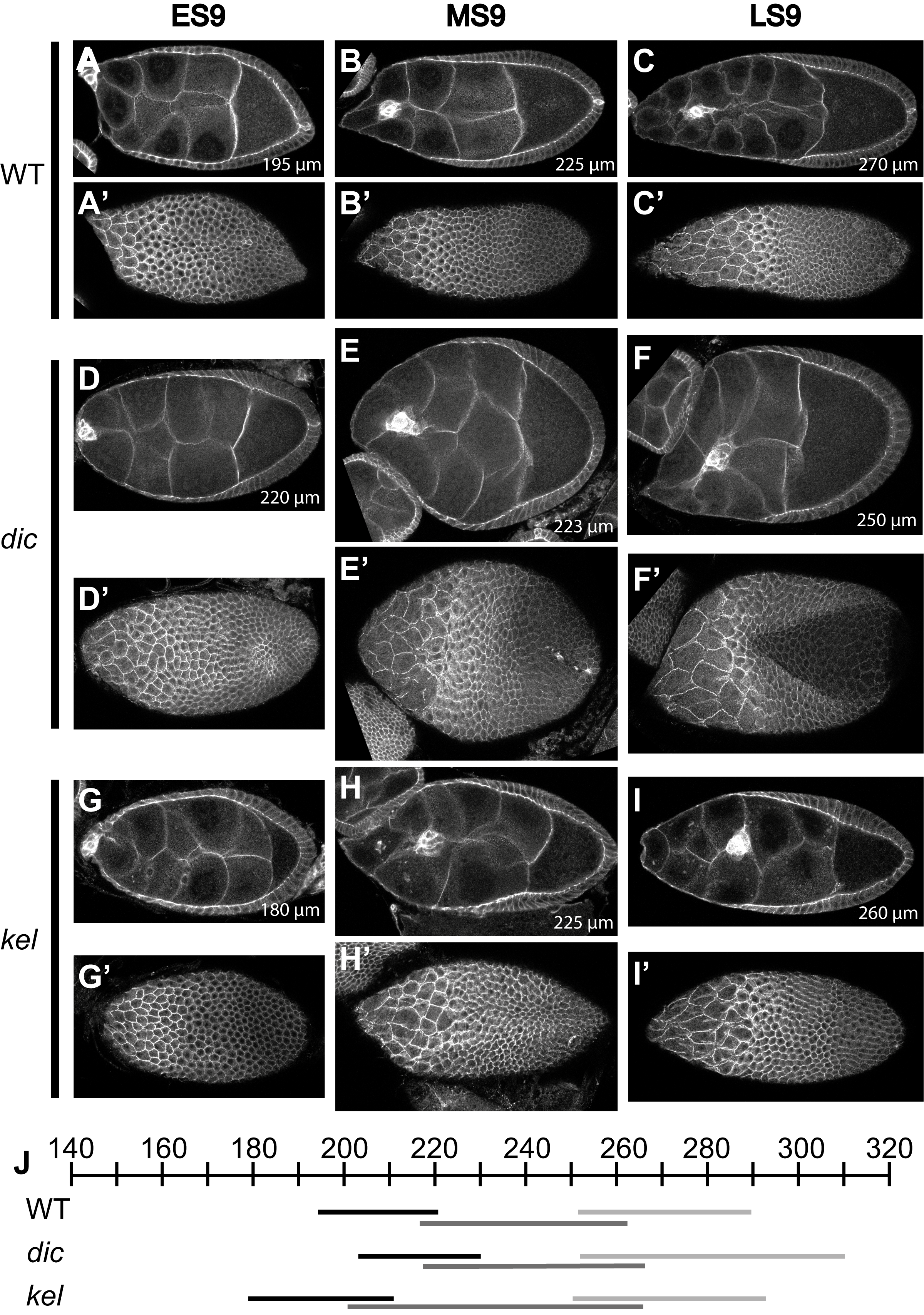

Supplement: S5 Fig — (A–I) Follicles of ES9, MS9, or LS9 in WT, dic, or kel follicles. For each stage and mutant, a section through the middle of the follicle and a projection of all the sections where StCs are visible are presented. Follicle lengths are indicated. (J) Correspondence between developmental stages (ES9, black; MS9, dark gray; LS9, light gray) and follicle length. dic, dicephalic; ES9, early S9; kel, kelch; LS9, late S9; MS9, mid S9; StC, stretched cell; WT, wild type. (TIF) [file pbio.3000940.s008.tif]

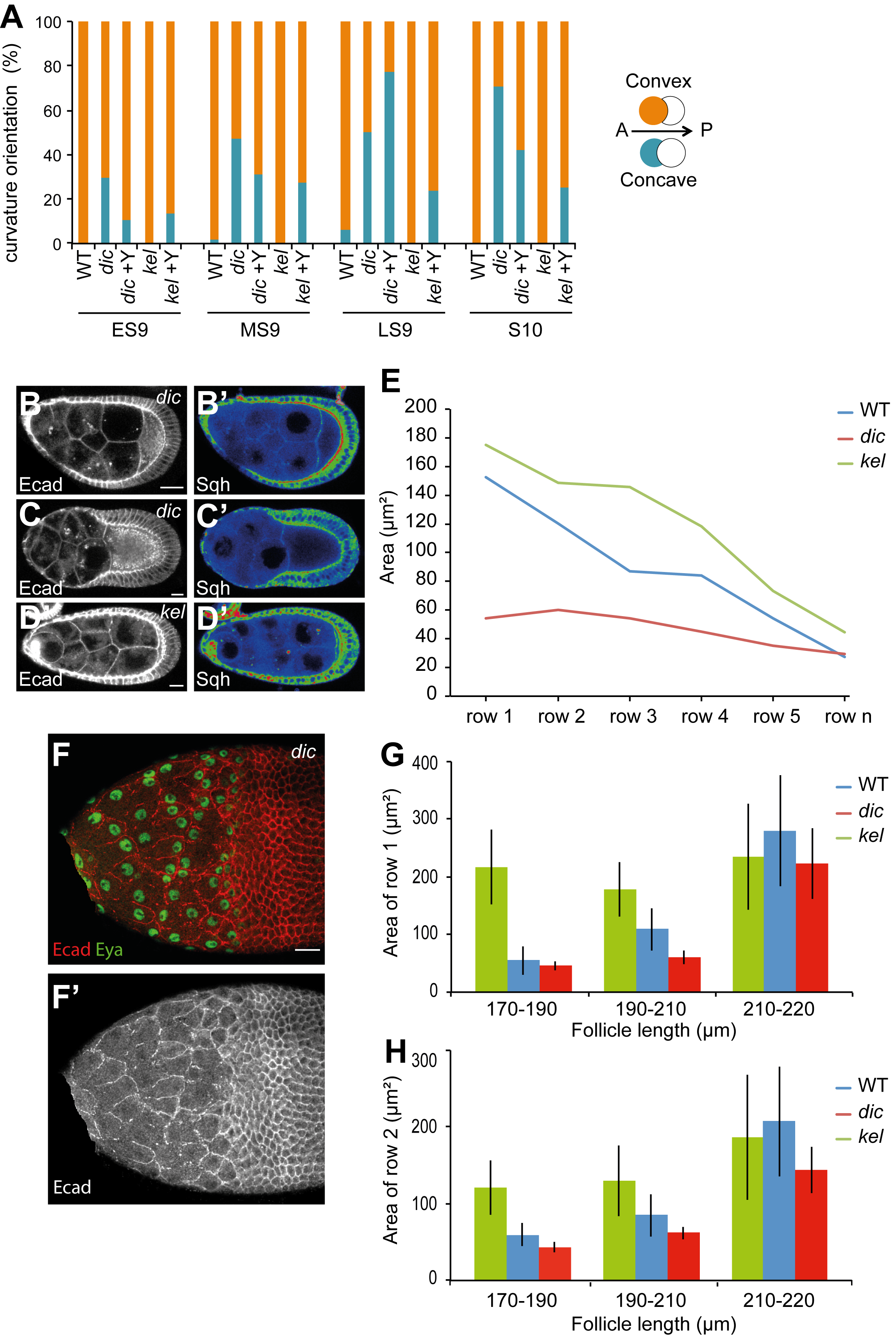

Supplement: S6 Fig — (A) Curvature orientation in WT, dic, or kel follicle during S9 and S10 in presence or not of the ROCK inhibitor (Y-27632) (n > 10 follicles per stage). (B–D) Sections through dic live follicles (B–C) or kel live follicles (D). (E) Apical StC area along the antero-posterior axis, from row 1 (anterior) to row n (posterior), of WT (blue), dic (red), and kel (green) MS9 follicles (n > 10 cells for each row). (F) Projection of all the sections where StC are visible in a S10 dic follicle. Most of the AJs are still visible (F’). (G, H) Apical StC area of row 1 (G) or row 2 (H), in function of WT (blue), dic (red), and kel (green) follicle length (n > 10 cells for each row). Scale bar: 20 μm. Data for graphs (A), (E), (G), and (H) can be found in the S1 Data file. AJ, adherens junction; dic, dicephalic; kel, kelch; MS9, mid S9; S, stage; StC, stretched cell; WT, wild type. (TIF) [file pbio.3000940.s009.tif]

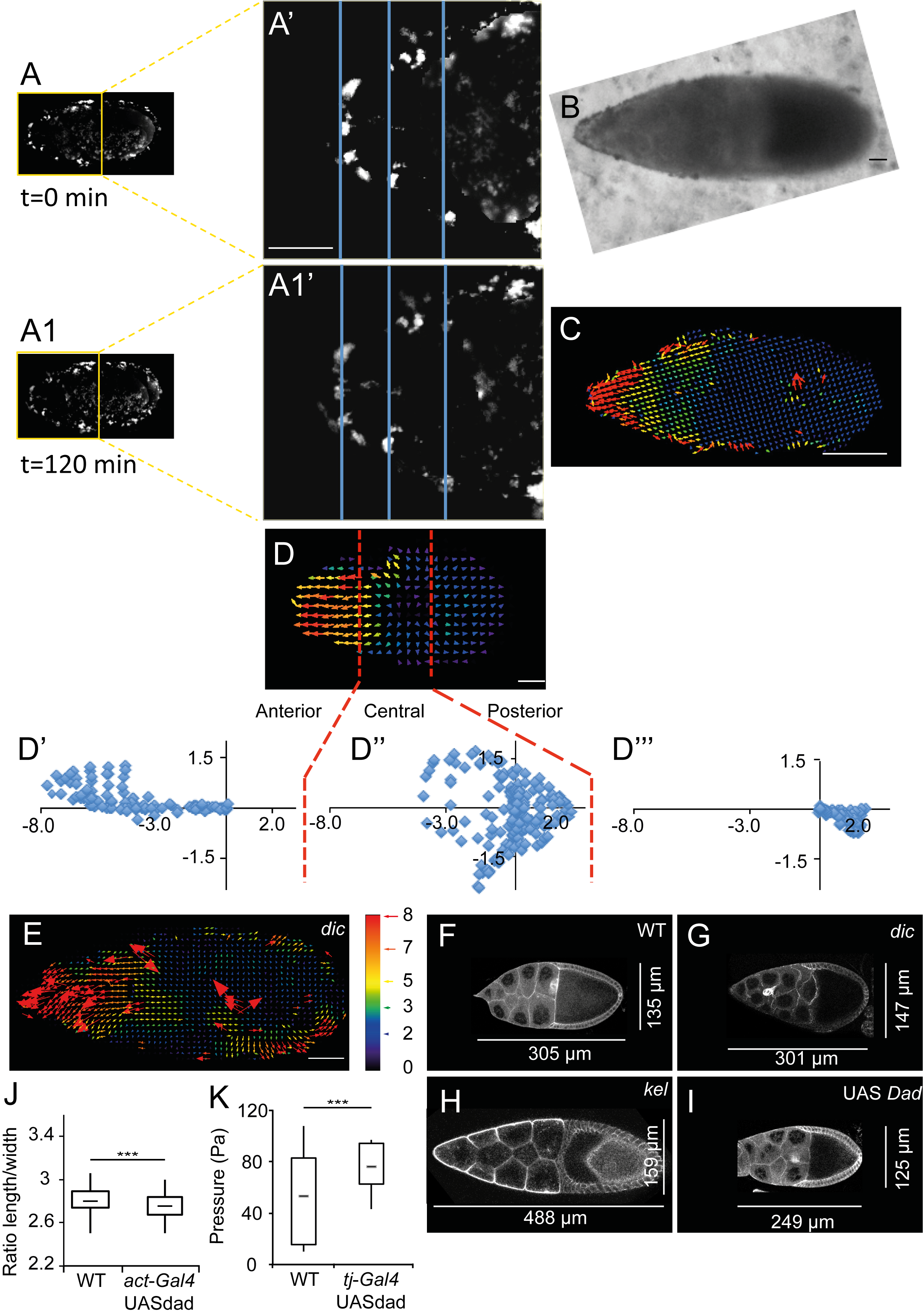

Supplement: S7 Fig — (A) LS9 follicle with fluorescent beads at two different time points (A, A1). (A’) and (A1’) are enlarged views of the yellow boxes drawn in (A) and (A1), respectively. (B) The S9 follicle presented in (A). (C) PIV representation from the beads positioned on the follicle presented in (A). (D) PIV representation from the beads positioned on an ES9 follicle and plots presenting the coordinates of the vectors for the anterior (D’), central (D”), and posterior (D”‘) regions. For each area, the initial (x, y) coordinates of the beads are (0,0). Each blue dot corresponds to the final x and y coordinates (in μm) of a bead. I PIV representation from the beads positioned on a S9 dic follicle. (F, G, H, I) LS9 follicles from WT (F), dic (G), kel (H), or Dad-expressing (I) females with the length and the width of the follicles indicated. In (I), UAS-Dad is expressed under the tj-Gal4 driver. In (H), the follicular epithelial above the oocyte is collapsing in the oocyte, possibly because of the lack of pressure in the latter. (J) Egg shape from WT females or females expressing Dad in the follicular cells under the actin promoter (large Flip-out clones) (n > 40 per genotype). (K) Pressure in anterior nurse cells in WT and in Dad-expressing follicles at MS9. UAS-Dad is expressed under the tj-Gal4 driver. Scale bars: 20 μm. Data for graphs (J) and (K) can be found in the S1 Data file. dic, dicephalic; ES9, early S9; kel, kelch; LS9, late S9; MS9, mid S9; PIV, particle image velocimetry; S, stage; WT, wild type. (TIF) [file pbio.3000940.s010.tif]

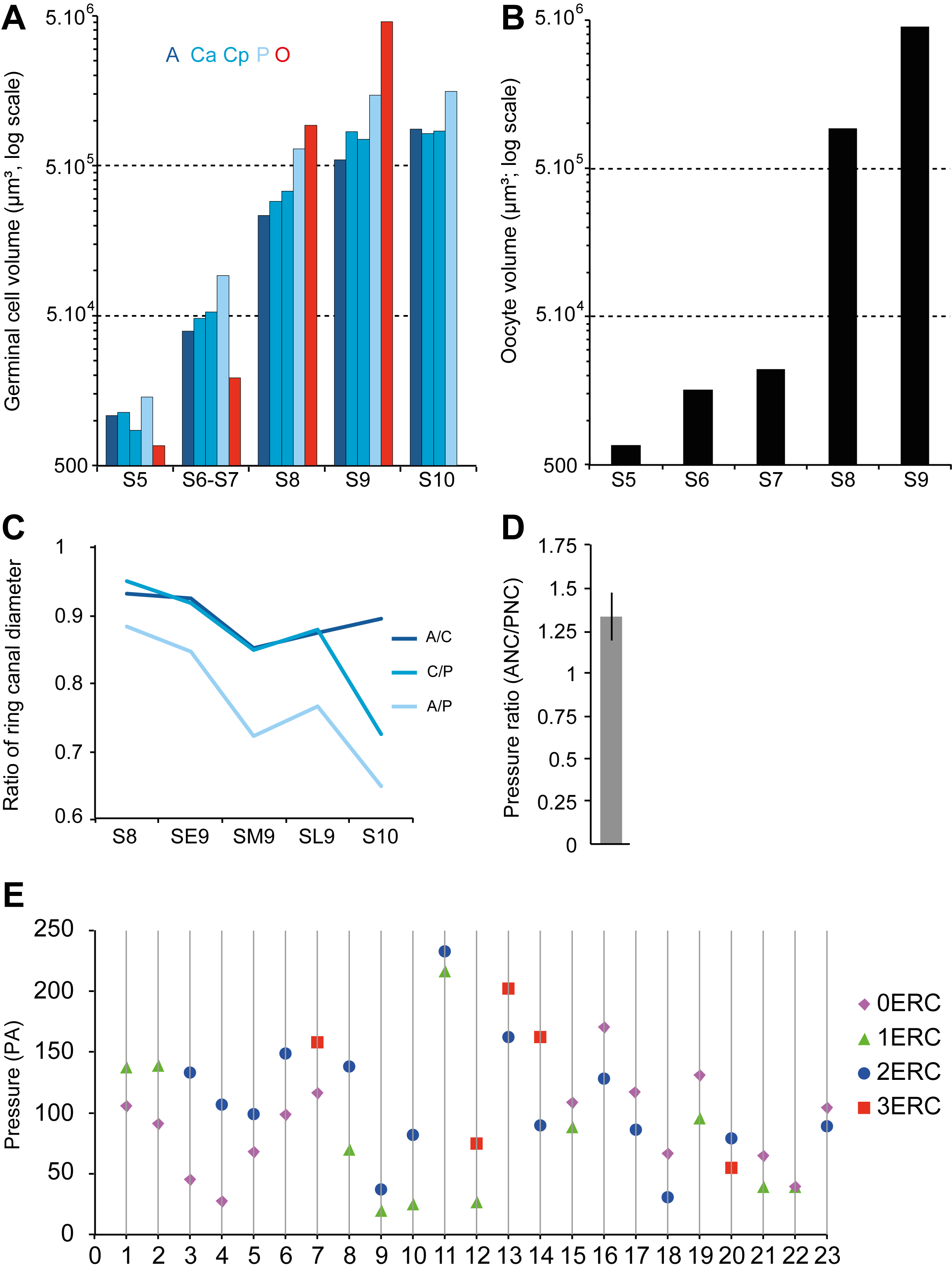

Supplement: S8 Fig — (A) Volumes of anterior (“A,” dark blue), central (“C,” cyan), and posterior (“P,” light blue) NC and the oocyte (red) from S5 to S10 WT follicles. Central NC are further subdivided into those abutting the Ca and those abutting the four Cp. (B) Volume of the oocyte from WT S5 to S9 follicles (n > 2 per stage). (C) Ratios of RC diameters at different follicular stages. Ratios are presented for A/C NC, A/P NC, or C/P NC. (D) Ratio of pressure between the ANC and the oocyte-connected PNC 1RC NCs (n = 4). (E) Inner pressure in two posterior NCs in WT S10 follicles. The number of ERCs of the two probed NCs is indicated by a symbol (diamond, triangle, circle, or square). Data for graphs (A), (B), (C), (D), and (E) can be found in the S1 Data file. 1RC, single RC; A/C, anterior versus central; A/P, anterior versus posterior; ANC, anteriormost NC; C/P, central versus posterior; Ca, anterior NC; Cp, posterior NC; ERC, entrance RC; NC, nurse cell; PNC posteriormost NC; RC, ring canal; S, stage; WT, wild type. (TIF) [file pbio.3000940.s011.tif]
