# Supplementary material for: Gradient in cytoplasmic pressure in germline cells controls overlying epithelial cell morphogenesis
Source: PLoS Biol. 2020 Nov 30;18(11):e3000940. doi: 10.1371/journal.pbio.3000940 (PMC7703951; doi:10.1371/journal.pbio.3000940)
Supplement: Attachment — Submitted filename: Response to Reviewers.docx [file pbio.3000940.s012.docx]

**Response to reviewers**

We would like to thank the referees for their constructive comments and suggestions. To address these, we have made numerous revisions to the manuscript and the figures, which we feel have helped clarify and strengthen most of the conclusions. Below are our detailed replies (in blue text) to each of the points raised.

**REVIEWER 1**

*In the manuscript by Lamire et al., the authors explore the role of cytoplasmic pressure in morphogenesis of the Drosophila follicle. Specifically, how mechanical changes in nurse cells (NCs) lead to the flattening of the epithelial layer surrounding the follicle and the development of "stretched cells" (Stcs), a squamous epithelium, in the anterior part of the follicle. This process occurs along with the elongation of the follicle in the anterior-posterior direction (AP). Previous work from other labs had identified a role for the basal membrane (BM), laid down in parallel rings as the whole follicle rotates along the AP axis. While it has been shown that the BM is required for the development of ellipsoid follicles, it is unclear how the shape is generated, leading researchers to hypothesize that internal pressure generated by the expansion of NCs acts together with the confinement of the BM to produce the characteristic ellipsoid shape of the follicle.*

*In this manuscript Lamire et al used atomic force microscopy (AFM), genetic manipulations and light imaging to probe the system. Using a limited set of genetic manipulations, the authors firt concluded that the asymmetry in the follicle arises from mechanical features. Then, using AFM they were able to detect differences in stiffness along the AP axis that they were able to correlate with the differential flattening of Stcs in the anterior follicle as well as with the posteriorly orientation of cell membrane curvature in NCs. They then used experimental values of follicle curvature and stiffness to calculate the internal pressure of NCs, assuming a direct relationship and no interference from the BM or the Stcs, to conclude that a gradient of internal pressure exists in NCs and that this gradient plays an important role in the asymmetrical morphogenesis of the follicular epithelium. They then tested their model by the performing AFM measurements and morphological analyses together with genetic manipulations that alter key aspects of follicle development, and also correlate their observations with insights into ring canal characteristics.*

*Overall, this is an interesting, thought provoking and well written manuscript that is built around a logical and plausible scenario that reaches interesting conclusions that may be of broader significance. The conclusions are very interesting and likely correct at a basic level, i.e. that the pressure created by NCs is critical for the morphogenesis of the follicle. However, the authors used a technique (AFM) that provided a minimally invasive, yet indirect method and they also made a number of assumptions that, while reasonable, were not sufficiently tested and validated experimentally. Particularly, they assumed that the AFM data reflects the properties of NCs while the elaborate BM and the Stcs are in between the probe and the NCs.*

*To better support their approximation and their conclusions the authors should:*

***1-Perform a number of physiological manipulations including hypo and hyperosmotic media (using various osmolites, not just 1 M NaCl addition). 1a-Do different NCs respond to osmotic manipulations differently?***

We now present AFM measurements on follicles treated with different osmolytes (NaCl and Sorbitol) and different concentrations for NaCl (0.5M and 1M). We repeatedly observed a reduction of pressure (Figure S2G). We also did not observe any differences with 1M NaCl as a function of NC position. We indicate this in the legend.

|  | **Figure S2G:** Fold-change of inner pressure following osmotic treatment shown as a function of solution osmolarity (NaCl 0.5M in water – NaCl 0.5M in PBS – Sorbitol 1M in PBS – NaCl 1M in PBS from the lowest to the highest osmolarity). No significant influence of the position of the NC (anterior, central or posterieur) has been observed. |
| --- | --- |

**Changes in the manuscript:**

Page 10: “In addition, we performed different osmotic treatments and always observed a sharp decrease in stiffness values, demonstrating that AFM measurements are responsive to osmotic changes (Figure S2E, S2F, S2G). “

***2-Test how changes in the extracellular matrix affect AFM measurements and possibly the pressure inference.***

**Fig. S2H:** AFM measurements of inner pressure in A, C or P nurse cells after collagenase treatment.

We have now carried out AFM measurements on follicles after collagenase treatment (Figure S2H). As the treatment leads to nurse cell expansion, we hypothesized that NC cytoplasmic pressure is lowered compared to WT follicles. As expected, NC display lower pressure compared to untreated samples, but the gradient is still present from anterior to posterior. Pressure decreases by a factor of 4.3, 5.8 and 8.3 for anterior, central and posterior NC, respectively.

**Changes in the manuscript:**

Page 10: “Finally, it is known that follicles are surrounded by a stiff basement membrane [31,51]. To determine the importance of the BM in nurse cell pressure, we also performed AFM measurements on collagenase-treated follicles. In such follicles, the antero-posterior pressure gradient is still detected, although the absolute values of pressure are lower than in untreated follicles, possibly due to NC expansion (Figure S2H).”

**3-Test more extensively the contribution of the cell cortex and the follicular epithelium to the AFM data.**

|  | **Fig. 2D:** AFM measurements of inner pressure in WT late stage 9 and stage 10A follicle before and after Latrunculin B treatment. |
| --- | --- |

We now present AFM measurements before and after latrunculin treatment on WT follicles (Figure S2D). A mild reduction is observed, indicating that Actin organisation is not the predominant determinant of the NC mechanical properties.

**Changes in the manuscript:**

Page 9: “In order to assess this, we performed measurements before and after the addition of Latrunculin B, which disrupts actin cytoskeleton or of the pharmacological ROCK inhibitor (Y-27632), which has been shown to inhibit Myosin activity [49,50]. We observed that a mild decrease in pressure is observed after incubation in Latrunculin B (Figure S2D) and that blocking Myosin II decreases pressure by a factor of 1.6 for both anterior and posterior NC (Figure 2D).”

**Other points:**

**4-Given that the curvature of the follicle depends on the overall geometry of the tissue, which depends on the BM and other features, can the curvature of the follicle really be used to infer the pressure of in NCs? In other words, is this really a local value the way it is determined? Here a more elaborate demonstration and theoretical validation is needed.**

To address this comment, we have added a Methods subsection titled “Justification of pressure deduction from AFM”. Briefly, collagenase treatment shows that the BM constrains the NC shape. Based on the mechanical model in Ref. 100, on follicle geometry, and on our indentation depths, the measurement scale is about 10 µm, which is smaller than the size of a nurse cell and so is well suited to obtain the value of pressure for these underlying cells.

**Changes in the manuscript:**

Page 30: New subsection “Justification of pressure deduction from AFM”

**5-Could the posterior orientation of membrane curvature in NCs result simply from developmental timing? i.e. the anterior NCs expand first and so on.**

To answer this question, we now present a graph (Figure S4E) that shows posterior membrane curvature as a function of NC position along the A/P axis from stage 5 (S5) to stage 10. We observe that the percentage of convex curvature increases at S8 whatever the position of the NC and remains high during early S9. At late S9/S10, almost all posterior membranes of the posterior NC are convex; the percentage of convex posterior membranes of the anterior and central NC decreases at this stage, with only about 10% concave. These data indicate that the posterior orientation of membrane curvature in NCs does not result from developmental timing. They also confirm that the oocyte has a significant gradient in pressure until S8 since almost none of the posterior membranes of the posterior NC are convex at stage 5, 6 and 7.

Please note that in this version of the manuscript, all graphs (Figure 1H, Figure S4E, F and G) present data in percentage (except for the curvature of the anterior membrane of the oocyte), since we realized that our representation in real number was confusing.

**Changes in the manuscript:**

Page 11: “We observed that posterior NC membranes become progressively convex during S8 and S9, with no significant influence of the position of the membrane along the A/P axis (Figure 2G, 2H and Figure S4B, S4C, S4E).”

|  | **Fig. S4E:** Percentage of convex (orange), flat (grey) and concave (blue) NC posterior membrane curvatures in function of the position of the nurse cells along the A/P axis (Anterieur (A), Central (C) and Posterior (P)) in WT stage 5 to stage 10 follicles (n comprised between 9 and 49 cells). |
| --- | --- |

**6-How does the initial elliptical geometry impact how the internal pressure acts on the structure? Perhaps some discussion point could be added.**

The ellipsoidal geometry is fully taken into account by the model used for pressure deduction, through the values of the two radii of curvature. This is now made explicit in the new subsection “Justification of pressure deduction from AFM”.

**Changes in the manuscript:**

Page 30: New subsection “Justification of pressure deduction from AFM”

**7-Hydrostatic pressure is isotropic, how can there be a gradient of pressure with an enclosed structure? Here, a more elaborate concept needs to be introduced to account for the proposed model. 7b-Similarly, what prevents the NCs with higher pressure to expand at the expense of the other cells? Are ring canal features sufficient?**

We have added a paragraph in the discussion to address this comment. Briefly, it was recently predicted and tested in plant tissues that gradients in pressure may be maintained, provided that water and cytoplasmic movement is partially limiting for cell volume changes (Cheddadi et al. PLoS Comput Biol 2019; Long et al. Curr Biol 2020). We expect the situation to be similar here, though testing this hypothesis is beyond the scope of the present study.

**Changes in the manuscript:**

Page 21: “Our observation of a pressure gradient raises the question of how it can be maintained without certain nurse cells growing at the expense of others or emptying their contents through RC. A similar question was recently investigated theoretically and experimentally in plant tissues [60,61]: plasmodesmata and the cell wall play the same role as RC and the actomyosin cortex, respectively. Extrapolating from those studies, it would be expected that nurse cells maintain their relative volumes whenever actomyosin cortex viscosity and RC hydraulic conductivity are low enough, so that the movement of water and cytoplasm is partially limiting changes in cell volumes.”

**8-What is the origin of NC pressure? Are there channels that could be manipulated?**

In 2015, Kruger and Bohrmann described the various bioelectric signals (membrane-potential gradients, ionic current patterns or domains of differing intracellular pH) that may be present during oogenesis. In particular, they described the spatial distribution of amiloride-sensitive Na+,H+-exchangers (NHE) and Na+-channels. It has been shown that NHE are important for cell volume regulation in *Drosophila*. For instance, the amiloride-sensitive Na+-channel dGNaC1, which is expressed in gonads, may be involved in water uptake during the final maturation of the oocyte. Kruger and Bohrmann show that the Amiloride-sensitive Na+-transporters are distributed in an anteroposterior gradient within the NC group, with more transporters in anterior NC compare to posterior NC, but they state that the staining (through the fluorescent inhibitor amiloride-FL) is so strong that it precludes the observation of specific membrane labelling.

We agree that understanding the origin of the NC pressure is important. Genetically or chemically manipulating these canals appear to be feasible, though getting the right conditions to generate mild effects and avoid follicle death is likely to be challenging. In this new manuscript, we now discuss these channels and their putative involvement in cell volume control.

**Changes in the manuscript:**

Page 21: “Difference in the spatial distribution of amiloride-sensitive Na+,H+-exchangers (NHE) and Na+-channels are also likely to play a role in controlling NC volume. In 2015, Kruger and Bohrmann described that the Amiloride-sensitive Na+-transporters are distributed in an anteroposterior gradient within the NC group, with more transporters in anterior NC compare to posterior [59]. Although drastic modifications of such channel activities might be deleterious for follicle integrity, analysing NC volume differences in conditions mildly affecting their activity could be informative.”

**Minor points**

**9-Can the authors exclude that the variations in curvature they observe in Figure 1-H-J are not due to uneven digestion of the matrix?**

In Chlasta et al. (2017), we described the effects of collagenase on the BM (Figure 5, Figure S1 and Movie 1). The movie shows the NC and the oocyte becoming progressively rounder. Change in shape is visible for all cells 20 min after the collagenase treatment starts. No further major changes in shape are observed after 70 min. The figures in Chlasta et al. show that tears are present above the StC and halfway through the posterior part of the follicle with a major tear spanning the circumference of the follicle at the junction between the StC and the oocyte. In the present manuscript, experiments were performed after 80 minutes of treatment. We realized that we forgot to mention this duration. We now added it in Materials and Methods and mentioned that the experimental condition used are as described in Chlasta et al. (2017). In addition, we would like to point out that we previously demonstrated that the stiffness of the BM is higher above the oocyte compared to that above the StC at stage 10. Therefore, it is unlikely that the collagenase treatment allows in one hand the formation of round oocyte, but on the other hand be insufficient to remove the constraint above some NC.

**Changes in the manuscript:**

Page 31: “The reactions were stopped with 10mM L-Cystein after 80 min of incubation. These experimental conditions lead to collagen digestion all around the follicles (see Chlasta et al., 2017 ([31]) for details).”

**10-Do different NCs respond differently to osmotic treatments under the conditions used in Figure 1 (i.e. collagenase treatment).**

|  |  |
| --- | --- |
| **Before NaCl treatment** | **After NaCl treatment** |

We did try to monitor NC shape changes in those conditions (collagenase treatment followed by an osmotic choc), but they were too harsh to detect differences in NC responses. The images presented below show a collagenase-treated S10 follicle, before and after the addition of NaCl. As it is difficult to draw any firm conclusions from these data, we have not included them in the manuscript. The follicles are very fragile after this treatment, preventing any further manipulation.

**Very minor points:
the authors use "Pression" in figures, do they mean "pressure"**

We have corrected this typo in Figure 1 and in Figure 7.

**line 231: should be "compared"**

We have corrected this typo.

**Reference 78 should be "Adams, Keller and Koehl". This is a truly insightful and seminal paper that should be cited here, but it did not show that pressure leads to axis elongation, that was actually done by Ellis et al (PMID 23460678).**

We thank the reviewer for bringing the Ellis et al paper to our attention. We have added it to the manuscript (Page 23) and corrected the “Adams (1990)” citation.

**REVIEWER 2**

This is an extremely original study and a fascinating question. This work addresses the complex question of how the mechanical properties of one group of cells influences the properties of neighboring cells, and how this interaction shapes the final organ these cells are part of. This work is not without conceptual and technical difficulties, intrinsic to the complex question being addressed, and I think the authors do a great experimental work.

**Major points:**

**1. Intrinsic to a complex question being studied, I think one has to be careful on the conclusions, and this is one of the main problems with the manuscript. Often the authors conclude a causal relationship when they should only conclude that there is an important correlation, which may be causal or not. There are a few examples of this problem, where I think the authors over-interpret results and make statements that are not always fully supported by the data. For example, the order of events is difficult to prove: e.g. line 405 says that 'These together (NC pressure and FC shape changes) sculpt the final organ, the egg, by indirectly acting on the mechanical properties of the basement membrane'. However, the authors have not shown this to be the case. There is no data demonstrating that changes in the BM are due to the NC pressure and the FCs flattening…there is a correlation but not a causal effect. Another example: Fig1H-I: Could the collagenase differential effect be related to differences in BM composition/sensitivity to treatment? And not due to differential NC cytoplasmic pressure?**

**Also, Line 302: Title 'High cytoplasmic pressure, and subsequent StC flattening, leads to enhanced anterior follicle expansion'… this title is misleading...the experiments here show that flattening and/or BM softening contribute to anterior expansion.**

We agree with the reviewer that several times in the earlier version of our manuscript, we tended to add correlations from certain experiments to conclusions from other data, which was indeed confusing. We have now carefully scanned the manuscript and modify such instances to bring them closer to the experimental results.

**Changes in text:**

Page 14: Title: “High NC cytoplasmic pressure and TGFß signalling lead to enhanced anterior follicle expansion.”

Page 15: “From this, we propose that BM softening and StC flattening are important to maintain NC integrity, likely by promoting anterior expansion and maintaining NC pressure under a certain threshold.”

Page 19: “Here, we show that cytoplasmic pressure is graded within a well-defined group of nurse cells and that this pressure controls TGFß expression in the surrounding epithelial cells. As TGFß itself induces their shape changes and BM softening, we propose that *in fine*, both NC cytoplasmic pressure and BM softening promote enhanced anterior follicle elongation (Figure 7).”

**Another example: Fig1H-I: Could the collagenase differential effect be
related to differences in BM composition/sensitivity to treatment? And not due to differential NC cytoplasmic pressure?**

In Chlasta et al. (2017), we described the effects of collagenase treatment on the BM (Figure 5, Figure S1 and Movie 1). The movie shows the NC and the oocyte becoming progressively rounder. Shape change is visible for all cells 20 min after the collagenase treatment starts. No further major change in shape is observed after 70 min. Tears are visible above the StC and halfway through the posterior part of the follicle with a major tear spanning the circumference of the follicle at the junction between the StC and the oocyte. In the present manuscript, experiments were performed after 80 minutes (which we had earlier forgotten to mention). This has now been added to the Materials and Methods and we further clarify that the experimental condition used are as described in Chlasta et al. (2017). In addition, we would like to point out that we have previously demonstrated that BM stiffness is higher above the oocyte than above the StC at S10. Therefore, it is unlikely that the collagenase treatment allows the formation of round oocytes on the one hand, but is insufficient to remove the constraint above some NC on the other.

**Changes in the manuscript:**

Page 31: “The reactions were stopped with 10mM L-Cystein after 80 min of incubation. These experimental conditions lead to collagen digestion all around the follicles (see Chlasta et al., 2017 ([31]) for details).”

**2. The authors use various original and valuable techniques to measure stiffness and pressure of the tissue/cells. One of these techniques is AFM. This approach is valuable, and the results obtained I believe are robust, but I fail to see how the AFM values are directly linked to only NC cytoplasmic pressure to the point that the authors say 'demonstrates the presence of a cytoplasmic pressure gradient …" line 209…aren't the BM and the FCs also contributing to any values obtained with AFM? At this point the authors cannot say 'demonstrate' and only when put together with other results, then they could say there is strong data suggesting…**

We now provide an additional subsection “Justification of pressure deduction by AFM” to address the mechanical contribution of the BM and of the StC during AFM measurements. We agree that detecting changes in AFM measurements is not sufficient on its own to claim the presence of a gradient of pressure in the NC compartment and that the demonstration of its presence requires alternative measurements, such as the curvature of the posterior membrane. We have now tempered the conclusion of this paragraph. Please note that we also now present AFM measurements after collagenase treatment and that we observed the presence of a gradient in this condition too.

**Changes in the manuscript:**

Page 30: Additional subsection “Justification of pressure deduction by AFM”.

Page 10: “Finally, it is known that follicles are surrounded by a stiff basement membrane [31,51]. To determine the importance of the BM in nurse cell pressure, we also performed AFM measurements on collagenase-treated follicles. In such follicles, the antero-posterior pressure gradient is still detected, although the absolute values of pressure are lower than in untreated follicles, possibly due to NC expansion (Figure S2H). Overall, the data are in favor of the presence of a cytoplasmic pressure gradient within the NC compartment at late stage 9 and at stage 10.”

**3. Another tricky part, which I guess is a limitation of the system or the approach, is the fact that the gradient of pressure should start at st8-early9, for it to be functional connected with flattening of FCs. But most measurements are later than that. Could the authors explain why more measurements are not taking place at st8, early st9, and most data presented is st9-st10? What are the limitations for measuring the pressure at earlier stages?**

Measuring the apparent stiffness of NC requires deforming both the BM and the StC. AFM measurements typically allow a maximum of 3-4 µm indentation. The thickness of the BM is on average 0.2 µm, whereas FC height varies from about 5 µm before S9 to < 1 µm after flattening. Therefore we cannot probe NC prior to S9. This is explained in Materials and Methods, in the section on the “bespoke protocol to probe internal NC pressure”.

**4. The last point that I found hard to get my head around, and that I think affects all experiments, is the staging of the follicles. How was staging exactly defined? The authors say various parameters were used to define the stage of the follicles, but how exactly? I think the authors need to be much more precise on how each stage is quantified, for each mutant and for each experiment.**

Fig S5: Staging of WT, *dic* and *kel* follicles

We thank the reviewer for pointing out that our description was incomplete. We now specify precisely how we sort *dic* and *kel* follicles in early, mid and late S9 (Materiels and Methods, and new Fig S5). For clarity, we have also removed most of the scale bars and have instead provided the actual length of the follicles.

**Changes in the manuscript:**

Page 26: “WT follicles were staged as a function of their full length (Lf), the oocyte length (Lo), and the timing of the different morphogenetic processes, including border cell migration and stretched cell flattening, that occur at stage 9 [25,26]. Usually, early stage 9 (ES9) follicles have an Lf of 205 ± 15 µm with row1 StC of about 100 µm^2^ of apical area; mid stage 9 follicles of 240 ± 20 µm with row1 StC of about 200 µm^2^ and late stage 9 follicles of 270 ± 20 µm with row1 StC of about 350 µm^2^. To stage *dic* and *kel* follicles, we first sorted them into early, mid or late stage 9 based on the progression of StC flattening. For *dic*, the start of ES9, MS9 and LS9 were ascribed based on the presence of StC with mild (about 50 µm^2^), medium (about 150 µm^2^), and severe (> 250 µm^2^) flattening, respectively. For *kel*, the start of ES9, MS9 and LS9 where ascribed when row1 StC showed mild (about 100 µm^2^), medium (about 200 µm^2^), and severe (above 350 µm^2^) flattening, respectively. We next plotted follicle length for each of the three groups to define the relationship between these two parameters (Fig. S5) and used both to stage the follicles. Accordingly, follicles with highly abnormal values of length in regards to StC flattening were excluded from our studies.”

**For example, line 273: 'quantification of StC apical surface area shows that dic mutant follicles start flattening later than WT…' how do you know this? The precise staging of both the wt and mutant follicles is essential to draw such a conclusion. This is just one example.**

Our staging protocol reveals that most *dic* follicles start showing some StC flattening when their length reaches about 215 µm, which is higher than the average for WT follicles. However, we agree that our phrasing was unclear at several places in the manuscript and we have now changed this.

**Changes in the manuscript:**

**Page 12: “**To prove this, we first analyzed NC membrane curvatures and observed that only half of the membranes bulge towards the posterior in *dic* follicles, whereas they are all convex in *kel* follicles (Figure 3A - 3C and Figure S6A; for staging of *dic* and *kel* follicles, see Materiels and methods and Fig S5).”

**Page 13:** “We first noticed that the average follicle length with early signs of StC flattening is higher in *dic* follicles and lower in *kel*, compared to WT (Figure S5, Figure 3J). For *dic* follicles, the gradient of flattening is strongly reduced and starts from row 2 (Figure 3G, 3H, 3K, and Figure S6E – S6H). In contrast, the flattening remains gradual in *kel* follicles, although it is less pronounced in the central area (Figure 3I – 3K, and Figure S6E – S6H).”

**In a similar manner, how do the authors know what is row1, 2, 3 etc...when the epithelia is disorganized, as it is the case in some mutants.**

We assigned rows manually. This difficulty often happens when inducing clones, which is not the case for *dic*. We rarely encountered difficulties to assign rows for *dic* follicles and never for the other mutations. In addition, data shows that StC areas in *dic* are usually not significantly different from one row to the next, indicating that even if few cells are misplaced, this does not affect the overall analysis.

**Other points:**

**When using AFM on the anterior NCs…how far is the probe from the anterior tip? The authors rightly mentioned that it is difficult to access the tip, so how far? And how consistent?**

We encountered this difficulty when probing some late S9 follicles (depending on the angle they form with the petri dish: the anterior extremity can either tilt up or down), and in all S10 follicles. In such cases, the most anterior cell is indeed unreachable, so we probe the region above the NC immediately posterior to the most anterior one. This means that the probe is usually able to reach an area located around 30 µm from the most anterior point. We have clarified this in Materials and Methods.

**Changes in the manuscript:**

Page 28: “The measurements referred to as “anterior” were not necessarily performed on the most anterior cell of the follicle, as the curvature of the follicle can sometimes prevent access to this cell. This difficulty occurs when probing certain LS9 (depending on the angle they form with the petri dish, with the anterior extremity tilted up or down), and most of the S10 follicles. In such cases, the probe is usually able to reach an area located about 30 µm from the most anterior point.”

**Reduced Myosin2 has no phenotype on progressive flattening, but treatment by the rock inhibitor shows defects….explain.**

We are not sure to fully understand this comment. We described in Grammont (2007) that reduced Myosin 2 impaired StC flattening (Figure 7E – generation of clones for an hypomorphic allele of *sqh)*. The treatment by the ROCK inhibitor will also likely induce abnormal flattening. We did not test this, as it would require couple of hours of incubation and as it is beyond the scope of this study.

**Also, regarding the possible role of actomyosin activity, I feel that the authors have not explored enough genetic options. There are several RNAis, DN and overexpression constructs that they could use for manipulating the activity of zipper and sqh.**

**Fig. S4I:** Percentage of convex (orange), flat (grey) and concave (blue) NC posterior membrane curvatures at different follicular stages in WT or in follicules with reduced *sqh* germline activity (n>167).

We thank the reviewer for this comment. We have now measured NC curvature in follicles expressing RNAi against *sqh* (two different lines were used: *sqh^HMS00830^* and *sqh^HMS00437^*) under three different germline-specific drivers (*matα4-Gal4*, *nos-Gal4* and *MTD-Gal4*). Similar percentages of convex curvature are observed for posterior NC curvature in four conditions (Fig. S4I). Two conditions (nos-Gal4 and MTD-Gal4) yielded flies without ovaries when the *sqh^HMS00830^* line is used.

**Changes in the manuscript:**

Page 11: “Note that the percentages of convex posterior NC membranes remain similar when RNAi against *sqh* is driven in the germline (Figure S4I).”

**dic was already used as a mutant to study FCs flattening, where it was shown for the first time that there is a link between NC growth and FC flattening. I think the authors do not introduce this early enough, and only present it briefly in one sentence after their own data (line 278: It also confims that NC growth impacts the degree of StC flattening as previously mentioned [32]…) I think this undermines the previous work.**

We cited this work in the introduction in the context of the role of the germline on StC flattening. To make this clearer, we have modified the relevant sentence in the introduction and added a sentence in conclusion.

**Changes in the manuscript:**

Page 4: “Finally, Kolahi et al. showed that StC flattening depends on the growth of the germline cells that controls the degree of flattening [32].”

Page 22: “Kolahi et al. had previously shown that the degree of StC size depends on NC growth [32].”

**Fig2. A. There is a gradient of tension from mid-to-late st9 between anterior and C, but not with P.**

We now show the statistical tests between A and P.

**Changes in the manuscript:**

Modified Fig. 2A.

**B. calculated how in this fig? AFM measurements? The authors say AFM measurements, but as mentioned above, this is probably not only reflecting NC cytoplasmic pressure. Similarly, in C) Schematic representation of mean cytoplasmic pressure in each region of WT S10…' not necessarily only of cytoplasmic pressure, but of AFM results, which probably include BM and FCs properties as well.**

We now provide an additional subsection entitled “Justification of pressure deduction by AFM” to explain that AFM measurements mostly reflect NC cytoplasmic pressure. We have also modified the captions of Fig. 2B and 2C to make it clearer.

**Change in text:**

Page 30: Additional subsection “Justification of pressure deduction by AFM”.

Legend 2A: “Box and whisker plot of cytoplasmic pressure deduced from AFM”

Legend 2C: “Schematic representation of mean pressure deduced from AFM”

**H: what are the values 0-100? % of cells with the type of curvature represented by colors?**

All graphs (Figure 2H, Figure S4E, F and G) were generated with exact numbers for each type of curvature, which we agree was confusing. We now present these data in percentages, except for the curvature of the anterior membrane of the oocyte, where the sample size is low.

**Change in Figures:**

Modified Figures 2H, S4E, S4F, and S4G; new Figure S4I

**- Fig3. Can you show membrane curvature of dic and kel mutants at the same stage than WT, and that being at early st9 for all cases, when the data is more relevant for the flattening process? Fig3 shows wt st10, and late st9 dic.**

|  | **Fig 3:** (A-C) Cross-section of WT (A; stage 8), *dic* (B; stage 8) or *kel* (C; early stage 9) follicles showing nurse cell membrane curvature. The length of the follicle is indicated in yellow. |
| --- | --- |

As advised by the reviewer, we now present S8 WT and *dic* follicles and an early S9 *kel* follicle. Follicles representative of the different parts of S9 are now presented in the new Fig S5.

**Change in Figures:**

Modified Figure 3: “(A-C) Cross-section of WT (A; stage 8), *dic* (B; stage 8) or *kel* (C; early stage 9) follicles showing nurse cell membrane curvature.”

**Fig3 title implies that the gradient controls flattening, but I fail to see this in the results presented in this fig. The stretching of dic and kel cells seems alike in fig H' and I', and both seem to have higher area than controls… in detail, Fig3k: The quantification of area says that dic has lower area than wt in arrows 1-5. I fail to see this in the representing image of dic on fig3H and H', where cells 2, 3 and 4 seem to have higher area than wt in G and G'.**

We agree with the reviewer that this was unclear due to our choice of scale bar. In fact, the WT follicle is actually smaller than the *dic* follicle. Follicles were chosen to facilitate visualisation of the presence or absence of a gradient of flattening. To make the presentation of these data less confusing, we now indicate directly on the images where follicle engths were measured, as well as the length of the follicles and the distance from row 1 to row 4 for each genotype. We have also improved the description in the legend.

**Changes in the manuscript:**

Caption of Fig 3G-H: “Note that most of the anterior *dic* cells have a similar apical area that the anterior WT cells of row 1 and 2, but for a much longer follicle length (the dashed yellow line corresponds either to the follicle length or to the length of the region of interest).”

**Line 273. Here the authors mention that there is no gradient of flattening, but there is still a small gradient, although reduced.**

The cells in row 1 are on average smaller than the cells in row 2 for *dic* follicles. This is not the case for WT follicles. A small gradient in *dic* is observed between rows 2 and 5. The text has been modified to explain this.

**Changes in the manuscript:**

Page 13: “We first noticed that the average follicle length with early signs of StC flattening is higher in *dic* follicles and lower in *kel*, compared to WT (Figure S5, Figure 3J). For *dic* follicles, the gradient of flattening is strongly reduced and starts from row 2 (Figure 3G, 3H, 3K, and Figure S6E – S6H).”

**Also in this section, line 274: 'In contrast, kel..' I partially disagree with this statement, as the ratio of kel mutant looks like dic mutant, with less pronounced gradient in the C region**

In *kel* follicles, there is a clear reduction in the apical area between rows 1 and 4 during StC flattening. The gradient is indeed less pronounced in the central region and this is now stated in the text.

**Changes in the manuscript:**

page 13: “In contrast, the flattening remains gradual in *kel* follicles, although it is less pronounced in the central area (Figure 3I – 3K, and Figure S6E – S6H)”.

**Fig4. Could the authors point out in the images in A-C what area is Ant, Central and Post here?**

We have included double arrows to delimit the different areas in the figure.

**Change in Figures:**

Modified Fig. 4.

**It would be nice to see an staining of the stages and ROI used for panel D. And, as mentioned above, this is one case where it is difficult to understand how the stages were defined here.**

The staging is now clearly explained in Figure S5, which also indicates follicle lengths. We have also included other WT and *kel* stages to illustrate panel D, which is now panel I.­

**Changes in the caption:**

Figure 4: “(A-L) Projections of the sections displaying pMad fluorescence in the StC in WT follicles (A, C, E, G, I, K), *kel* follicles (B, D, F, H) or *dic* follicles (J, L). To allow comparison of expression level between WT and mutants, the images were treated identically for each stage, but differently between stages (enabling comparison between A and B; C and D; E and F, J and K; L and M); G and H cannot be compared as intensity levels in H were too high to be enhanced as for G. The double white arrows on each follicle indicate areas above the anterior, central and posterior NC. The total length of the follicle is indicated. (M, N) Quantification of pMad fluorescence in WT and *kel* follicles (M) or in WT and *dic* follicles (N) in function of follicle length and stage (see fig. S5) (n > 15 nuclei per region).”

**Fig4e: can the authors show a pMad staining of a st7 wt to show that there is no pMad expression as stated in the text?**

**Fig: 4:** (A-L) Projections of the sections displaying pMad fluorescence in the StC in WT follicles (A, C, E, G, I, K), *kel* follicles (B, D, F, H) or *dic* follicles (J, L). To allow comparison of expression level between WT and mutants, the images were treated identically for each stage, but differently between stages (enabling comparison between A and B; C and D; E and F, J and K; L and M); G and H cannot be compared as intensity levels in H were too high to be enhanced as for G. The double white arrows on each follicle indicate areas above the anterior, central and posterior NC. The total length of the follicle is indicated.

We have now included a WT S7, where expression is weak and uniform in the anterior half of the follicle. Note that the *kel* follicle, which displays a clear staining, is smaller than the WT S7 follicle.

**Changes in Figures:**

Modified Fig. 4

**Also the quantification in fig4I shows no major differences in levels between the dic and wt of the same length, stats?**

We now provide statistics between WT and *dic* for the same length.

**Changes in Figures:**

Modified Figs. 4M,N

**Fig5. A' image has been heavily cropped around the egg chamber, why is that?**

Yes, the image is cropped to remove the beads present in the medium around the follicle. This was done manually in order to delimit, as precisely as possible, the follicle, as well as to eliminate a maximum of unrelated beads in order to facilitate data analysis. We now indicate this in the protocol and in the legend.

**Changes in text and in Caption:**

Page 33 and Fig 5A: “Beads outside the follicle were manually removed to simplify the analysis.”

**In B. what's the color scale representing? velocities?**

The color scale represents bead displacement in pixels over the course of the experiment. We now mentioned this in figure 5 and in the legend. We have also added the duration of the experiment in the legend.

**Changes in Captions:**

Fig 5B: “The vectors represent bead displacement (in pixel) and are color-coded as a function of their displacement. Duration of the experiment: 30 min.”

Fig 5G: “Duration of the experiment: 30 min.”

**In H-H''. the StC seem also broken? can the authors show an overlapping to see how the cells and the membrane breaks relate to each other?**

As far as we can tell, the StC are not broken. Ecad, Eya or GFP staining appear to be as expected. Careful analysis of the 3D image stack reveals no visible signs of damage. To make the figure clearer, we have now added the same three white arrows that were in Figure 5 H’’ to panels H and H’ as well.

**­**

**Changes in Figures:**

Modified Figs. 5H-H’’

**Fig6J. One of the important findings here could be the difference between A, C and P (at each stage), but these stats are not calculated. The calculated stats are only for RCs at the same area, at different stages.**

We now provide statistics between the different between A, C and P per stage.

**­Changes in Figures:**

Modified Figs 6J

**FigS6H, the kel follicle is showing a weird oocyte. What is that? Explain**

Almost all of the late *kel* follicles present circonvolution of the oocyte membrane and deformed epithelial layer (subsidence?). This is possibly due to the lack of pressure in the oocyte. We now indicate this in the legend of the figure.

**Changes in Captions:**

Fig S7H: In H, the follicular epithelial above the oocyte is collapsing in the oocyte, possibly due to the lack of pressure in the latter.

**Other points 2:**

**Line 97 revise, something is missing**

Done

**Line 133 revise, something is missing**

Done

**TkvA: is this CA working? Shouldn't you see at least an effect on the flattening timing, even if the progression is normal?**

Yes, when TkvA is expressed, the flattening starts earlier. We have described this previously (Brigaud et al., 2015). In Figure 1F of this manuscript, we can actually see a difference in the apical area between the TkvA-expressing cell (cell number 3) and WT cells located on the same row below it. However for the analyses in this manuscript, as we chose follicles based on the size of the clones (where most StC expressed TkvA), an internal comparison with WT cells at the same developmental stages is not always possible.

**Fig1. '(B) Adherens junctions (Ecad) remodelling …. Both parameters are marker of flattening (see movie 1)….' where is this movie 1 on flattening parameters? The movie 1 I saw does not cover this at all.**

We have now reformulated this.

**Changes in the caption:**

Fig 1B: “(To better visualise the shape and the position of different types of cells during flattening, see movie 1).”

**Line 429 talks about micropipette aspiration, but I failed to see these results**

We did not do micropipette aspiration, we just mentioned a paper that used this method.

**REVIEWER 3**

This is an extremely interesting paper on the mechanics of development in Drosophila, focusing on follicle development in Drosophila. The authors performed extensive experimentation, including AFM-based measurements of pressure in nurse cells, and demonstrated very clearly that thereexists a well-defined gradient in pressure from anterior to posterior that is closely linked with morphological changes in cells. The logic of the paper appears sound to me, and I think the work will be of great interest to developmental biologists of many kinds.

**The only points I would like to make is that the authors have consistently used the Laplace pressure Delta P = 2 gamma/R to deduce the pressure from measurements of cell wall radii of curvature R. First, the factor 2/R only holds for spherical interfaces (where the sum of the two principal curvatures is just 1/R+1/R=2/R). For a general interface it is the mean curvature that matters. Thus, I would like to know the precise 3D geometry of the interfaces that are seen as 2D projections in e.g. Figure 1H. Are these actually sections of spheres? Second, and more importantly, I would have thought that the authors would use their measurements of pressure and curvature to deduce the typical values of the surface tension. If I put in numbers, with Delta P ~ 150 Pa and R ~ 25 microns (2.5x10^-5 m), I find gamma ~ 2x10^-3 N/m ~ 2 dyn/cm~2 erg/cm^2, which for comparison is about 1/40th of the surface energy of water against air. Is this a sensible number? What does it tell us about the cells in this system? How does it compare with other systems?**

In fact, the two principle radii of curvature appear to have the same order of magnitude. Because of a more limited resolution in the direction of the optical axis of the microscope, curvatures outside of the 2D sections are not as precisely determined as within sections. We actually used an approximate argument assuming the two radii of curvature to be equal. We have now rewritten the relevant paragraph more clearly, noting mean curvature for non-spherical interfaces.

We thank the reviewer for the suggestion to estimate surface tension. We have now included this in a new Materials and Methods subsection “Justification of pressure deduction by AFM”. We observe a similar estimate of 5mN/m, which is consistent with values of cortical tension measured in isolated animal cells.

**Changes in the manuscript:**

Page 7: “structural elements, such as microtubules and non-cortical actin microfilaments (Figure 1J). We reasoned that the extent of curvature of the outward-facing NC membrane (the radius of the circle fitting this membrane) may reflect NC mechanics. Indeed, the radius of curvature (R) of a spherical interface is proportional to the ratio of its tension (γ) and inversely proportional to the difference in pressure (ΔP) between the two sides of the interface: R = 2γ/ΔP, which generalizes to non-spherical interfaces by replacing 2/*R* by the mean curvature of the interface [34,35]. Due to limited resolution along the optical axis of the microscope, we approximated NC mean curvature by 2/*R*, *R* being a radius measured in 2D optical sections along the A/P axis.”

Page 30: New section “Justification of pressure deduction by AFM”.
