# Supplementary material for: Gradient in cytoplasmic pressure in germline cells controls overlying epithelial cell morphogenesis
Source: PLoS Biol. 2020 Nov 30;18(11):e3000940. doi: 10.1371/journal.pbio.3000940 (PMC7703951; doi:10.1371/journal.pbio.3000940)
Supplement: Attachment — Submitted filename: Response to Reviewers.docx [file pbio.3000940.s013.docx]

**Response to reviewers**

We would like to thank the referees for their constructive comments and suggestions.
